# A Chapter in Dental History and Bibliography

**Published:** 1897-07

**Authors:** William H. Trueman

**Affiliations:** Philadelphia


### A CHAPTER IN DENTAL HISTORY AND BIBLIOGRAPHY.[1]

BY WILLIAM H. TRUEMAN, D.D.S., PHILADELPHIA.

Having been apprised of the fact that Dr. Kirk would give us to-night a *résumé* of Dr. Williams's work, exhibiting the latest achievements in the microscopic study of tooth-structure and of tooth-destruction by caries, I thought it might be of interest to take a glance backward, and have brought with me the works of Alexander Nasmyth, who was, I think I may safely say, the first of our profession to earnestly take up this line of investigation. Early in the thirties he began, as all careful and methodic investigators should, by first collecting, collating, and critically comparing

---

[1] Read before the Academy of Stomatology, Philadelphia, March 23, 1897.

all the accessible works of those who had preceded him, and thus made himself master of that which the world knew in this selected field. His industry and thoroughness in this preparatory work is commendable and well worthy of imitation. I show you a copy of Serres's work,[1] once the property of Mr. Nasmyth, with notes and pencillings made by him while prosecuting this study. The first results of his labors are embodied in this volume, "The Historic Introduction to Nasmyth's Researches on the Development, Structure, and Diseases of the Teeth," published in London in 1839. Its perusal will be a revelation to any one who imagines that dental literature prior to that date did not amount to much. Both in quantity and value it was such that no profession, however exalted, need hesitate to acknowledge it. I would that it were more accessible and better known.

Next, we have the beginning of his original work, this little volume of some sixty pages with nine plates, his "Three Memoirs on the Development and Structure of the Teeth and Epithelium," published in 1841; and lastly this, Nasmyth's "Researches on the Development, Structure, and Diseases of the Teeth," published in London in 1849, the conclusion of his labors, the manuscript of which he had barely completed when he was suddenly, in the midst of his usefulness, stricken down and incapacitated for labor. Valuable as this work is, it would undoubtedly have been more so had he been spared to superintend its publication.

These three volumes, the life work of Alexander Nasmyth, which has made the demonstration of this evening possible, were the beginning. The work of Dr. Williams shown us to-night by Dr. Kirk is the highest point so far reached as the result of sixty-five years earnest, continuous work of many enthusiastic laborers, who at every stage brought to bear the best that science and skill of the hour afforded in solving the mystery of tooth-building and tooth-destruction. In passing we may note for a moment that Nasmyth's time and Nasmyth's work may serve as a dividing line between the time when much that was accepted as truth rested upon theory alone, and the time when, as Nasmyth remarks, the advance in optical science rendered demonstration possible. Very early in his investigations Mr. Nasmyth was compelled to halt. The best microscopes and microscopic accessories attainable he found far too crude for the delicate work he had in hand. Enlisting the aid of expert opticians, partly by his suggestion and partly by his own mechanical

---

[1] L'Anatomie et la Physiologie des Dents, par A. Serres, Paris, 1817.

skill, he initiated a series of improvements which has made the microscope of to-day the marvellously precise instrument it is.

Eustachius wrote in the little book we examined together a few months ago, published at Venice in 1563, that if the germs of the permanent teeth are not seen in the fœtus, it is not that they do not exist, but that they are too small to be recognized. The microscope has enabled us to see with the eye that which this learned writer saw only in mind. Again, John Fuller, on page 55 of this little volume, "A Popular Essay on the Structure, Formation, and Management of Teeth," London, 1810, a work which, judging from the frequency it is referred to by his contemporaries, was held in high repute in the long ago, while discussing the phenomena of tooth-decay and its causation by some acidity of the surrounding fluids, cautiously suggests that the reason why it attacks certain spots in preference to others equally exposed, may be due to some congenital modifications of tooth tissue at that particular point. This haltingly made suggestion, Dr. Williams eighty-six years later demonstrated to be correct.

How slow, even in this progressive age, is real progress. It took some forty or fifty years of well-directed microscopic work to solve the mystery of dental caries. Nearly a decade and a half has elapsed since Dr. Miller announced that dental caries was the result of microbic energy, the initial stage, however, baffled his keenest scrutiny. Dr. Williams, by steady and patient work during the intervening years, aided by the improved facilities advancing science has supplied, seems to have completed the work, and we may now, for the first time in the history of our science, say that this riddle of the ages has been solved. So far, however, the stumbling block to the practical application of this discovery still remains. It seemed easy, after the discovery was made, to *prevent* instead of *repair* the ravages of dental caries. You may remember that Dr. Miller, and others, earnestly and hopefully began work to compass this desired object, only to find that an efficient germicide was also an equally efficient homicide. So long as this is the case, prevention remains a dream.

While we are looking backward permit me to call your attention to two other books I have with me. First, a well-preserved, handsomely bound copy of the "Elements of Odontology," by M. Lecluse, a surgeon dentist of Paris, dated 1754. Lecluse was a dental writer of well-deserved repute. This volume contains two of his works; the first is mainly upon the anatomy of the mouth and its surroundings, touching briefly upon some diseases of the gums and teeth.

I particularly call your attention to the second plate, page 131, representing three instruments devised by him for removing deposits from the teeth, especially a double-ended one, one end shaped for a push cut and the other for a draw cut. They are quite unlike the cumbersome instruments for this purpose so frequently figured in dental books. I have no doubt but that these instruments, devised by Lecluse nearly a century and a half ago, for delicacy and convenience would fully meet the approval of the most exacting of to-day, while his directions for performing the operation of cleaning the teeth, so far as the mechanical part is concerned, leaves little to be desired. Bound with it is a work he wrote a few years before, intended for the use of the public, on first and second dentition. It had been so well received both by the public and his professional brethren, he tells us, that to extend its usefulness he has included it in this work, intended mainly for professional readers. A careful study of dental literature fails to confirm the idea, so often expressed, that our profession has been remiss in the matter of instructing the masses in the care of the teeth.

This little work by Lecluse is but one of hundreds written by dentists with that object in view. To emphasize that fact I show you a copy of the very oldest dental publication at present known, possibly the only one in existence of so early a date, printed at Meyng, by Peter Jordan, August, 1532. It is entitled "Zene Arzney," and is a beautiful, well-preserved specimen of the antique German. Its scope and character is well shown by the title-page, translated as follows: "Teeth Medicine: against all kinds of defects and diseases of the teeth; many wholesome and well tried medicines extracted out of the books of Galen, Avicenna, Mesue, Cornelius Celsus, Pliny, and others; together with compendious and useful instructions as to how one can keep his teeth healthy, and how one can extract the bad hollow teeth, or their roots, which get to paining easily." A translation of a later edition will be found in the *Dental Cosmos*, vol. xxix., January, 1887, p. 1, and on page 68 of the same will be found a history of the copy there translated. In that history it is stated that when Mr. Crowley compiled his dental bibliography, in 1885, the only information of this book was a reference to it in a German medical journal issued about one hundred years ago, giving the date 1536, and place of publication Frankfurt. Shortly after the publication of Crowley's work a copy was found, and became the property of the *Dental Cosmos*. This was printed by Christian Egenolff, at the Leather Breeches (Ledderhosen), Frankfurt, 1541. The copy I show you bears the imprint of Peter

Jordan, at the "Leather Breeches," Meyng, 1532, nine years earlier, and Crowley's No. 15 is, I think, another edition under a somewhat different title, dated at Erfurt, 1614. In the Index Catalogue of the Army Medical Library at Washington, vol. xvi. p. 766, 1895, is noted a reprint, in 1891, by C. Weigler, of Berlin, of the little book I show you. So much for its history. For the contents I refer you to the translation in the *Dental Cosmos*, it is well worth reading, and old as it is may furnish a model for those who propose a series of popular treatises upon dentistry for the Sunday newspapers. The last admonition, if properly emphasized, might tend to make them popular,—"And finally, after eating, always wash the mouth with wine or beer." Further than it refers to filling cavities of decay with gold leaf, and thus carries back the use of gold for this purpose to at least 1532, this little book has value only as a curio, and as a well-preserved specimen of typographic art as it was three hundred and sixty-five years ago. It belongs to a class of publications of that period and later, of which a few specimens survive, devoted to matters of health and the toilet. Jordan and Egenolff were printers or publishers, not writers; the later name I find in connection with another work of a similar character, in Latin, of nearly the same date. In conclusion, from 1532 down to 1897, there never has been a time when publications giving popular instruction for care of the teeth were not accessible to the public; many of them have been well written, and, on a whole, in my judgment, those that I have seen compare favorably with the best of their time in any science or in any art.

